# SARS-CoV-2 Spike Protein Expression *In Vitro* and Hematologic Effects in Mice Vaccinated With AZD1222 (ChAdOx1 nCoV-19)

**DOI:** 10.3389/fimmu.2022.836492

**Published:** 2022-04-12

**Authors:** Richard Stebbings, Christopher Jones, Peter Cotton, Gillian Armour, Shaun Maguire, Vicky Skellett, Chi-Man Tang, Joanne Goodman, Tyler Brady, Virginia Takahashi, Andrew Daunt, Jean-Martin Lapointe, Taylor S. Cohen

**Affiliations:** ^1^ Oncology Safety, Clinical Pharmacology and Safety Sciences, BioPharmaceuticals R&D, AstraZeneca, Melbourn, United Kingdom; ^2^ Integrated Bioanalysis, Clinical Pharmacology and Safety Sciences, BioPharmaceuticals R&D, AstraZeneca, Cambridge, United Kingdom; ^3^ Research and Development, BioPharmaceuticals R&D, AstraZeneca, Macclesfield, United Kingdom; ^4^ Regulatory Toxicology and Safety Pharmacology, Clinical Pharmacology and Safety Sciences, BioPharmaceuticals R&D, AstraZeneca, Melbourn, United Kingdom; ^5^ Translational Medicine, Vaccines & Immune Therapies, BioPharmaceuticals Medical, AstraZeneca, Gaithersburg, MD, United States; ^6^ Microbiome Discovery, Vaccines & Immune Therapies, BioPharmaceuticals Medical, AstraZeneca, Gaithersburg, MD, United States; ^7^ Labcorp Early Development Laboratories Limited, Harrogate, United Kingdom; ^8^ Oncology Safety Pathology, Clinical Pharmacology and Safety Sciences, BioPharmaceuticals R&D, AstraZeneca, Cambridge, United Kingdom

**Keywords:** AZD1222 (ChAdOx1 nCoV-19), adenovirus-vectored vaccine, SARS-CoV-2 spike protein, COVID-19 vaccination, platelet and white blood cell parameters

## Abstract

Severe COVID-19 can be associated with a prothrombotic state, increasing risk of morbidity and mortality. The SARS-CoV-2 spike glycoprotein is purported to directly promote platelet activation *via* the S1 subunit and is cleaved from host cells during infection. High plasma concentrations of S1 subunit are associated with disease progression and respiratory failure during severe COVID-19. There is limited evidence on whether COVID-19 vaccine-induced spike protein is similarly cleaved and on the immediate effects of vaccination on host immune responses or hematology parameters. We investigated vaccine-induced S1 subunit cleavage and effects on hematology parameters using AZD1222 (ChAdOx1 nCoV-19), a simian, replication-deficient adenovirus-vectored COVID-19 vaccine. We observed S1 subunit cleavage *in vitro* following AZD1222 transduction of HEK293x cells. S1 subunit cleavage also occurred *in vivo* and was detectable in sera 12 hours post intramuscular immunization (1x10^10^ viral particles) in CD-1 mice. Soluble S1 protein levels decreased within 3 days and were no longer detectable 7–14 days post immunization. Intravenous immunization (1x10^9^ viral particles) produced higher soluble S1 protein levels with similar expression kinetics. Spike protein was undetectable by immunohistochemistry 14 days post intramuscular immunization. Intramuscular immunization resulted in transiently lower platelet (12 hours) and white blood cell (12–24 hours) counts relative to vehicle. Similarly, intravenous immunization resulted in lower platelet (24–72 hours) and white blood cell (12–24 hours) counts, and increased neutrophil (2 hours) counts. The responses observed with either route of immunization represent transient hematologic changes and correspond to expected innate immune responses to adenoviral infection.

## Introduction

The COVID-19 pandemic has produced substantial global morbidity and mortality, with more than 5 million deaths reported as of November 15, 2021 (1). Individuals with severe and critically severe COVID-19 commonly present with abnormal platelet parameters, including decreased platelet counts, compared with healthy individuals and those with mild/moderate COVID-19 (2). Poor coagulation outcomes including venous thromboembolism and arterial thromboembolism are associated with hospitalization and mortality from COVID-19 (3).

The SARS-CoV-2 structural surface glycoprotein antigen (‘spike protein’) has been observed to directly bind platelet Angiotensin-Converting Enzyme 2 (ACE2) receptors, enhancing platelet activation *in vitro* and potentiating thrombus formation *in vivo* (2). Cleavage of the spike protein S1 subunit (‘S1 subunit’) from host cells occurs during SARS-CoV-2 infection, and high plasma S1 subunit concentrations correlate with disease progression and respiratory failure in patients with severe COVID-19 (4). Due to its indispensable functions in mediating virus host-cell entry (5), the first wave of COVID-19 vaccine candidates were predominantly developed to target the spike protein, with several genetic vaccine platforms inducing its expression in vaccinees (6). Although gene-based spike protein vaccines have substantially reduced the risk of hospitalization and death from COVID-19 (7–10), it is unknown if vaccine-induced S1 subunit is similarly cleaved and present in the blood at high concentrations, and whether this has implications for the host immune response (11). There is also limited evidence on host immune responses or effects on blood parameters immediately following COVID-19 vaccination.

AZD1222 (ChAdOx1 nCoV-19), is a simian, replication-deficient adenovirus-vectored COVID-19 vaccine that is being used globally (1, 7), with >2 billion doses administered at the time of manuscript preparation. We conducted these experiments to test the hypothesis that S1 subunit is cleaved *in vivo* following AZD1222 immunization and to assess the potential effects of AZD1222 vaccination on host hematologic parameters.

## Materials and Methods

### *In Vitro* Assessment of Spike Protein Expression and Cleavage

#### Cell Culture, AZD1222 Transduction, and Cytotoxicity Assessment

**Figure 1:**
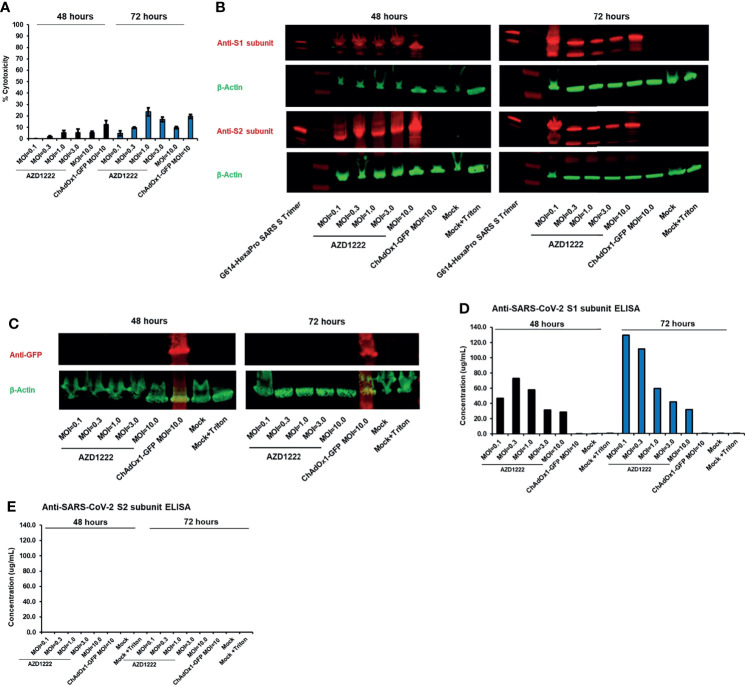
The S1 subunit of the SARS-CoV-2 spike glycoprotein is cleaved *in vitro* following AZD1222 transduction. **(A)** Viability of HEK293x cell lines 48 and 72 hours following transduction with AZD1222 at increasing input MOIs or ChAdOx1-GFP control. Error bars show the associated standard deviation for each sample. **(B)** Expression of SARS-CoV-2 S1 and S2 spike protein subunits 48 or 72 hours post transduction with AZD1222. **(C)** Expression of GFP control 48 or 72 hours post transduction with ChAdOx1-GFP. **(D)** Levels of SARS-CoV-2 S1 subunit and **(E)** full-length spike protein in cell culture supernatants at 48- and 72-hours post-transduction.

Human embryonic kidney (HEK) 293x cells (American Type Culture Collection) were grown at 37⁰C, 8% CO_2_, in FreeStyle™ 293 Expression Medium at a starting density of 1x10^6^ cells/mL. Cell cultures were transduced with AZD1222 at increasing multiplicities of infection (MOI); ChAdOx1-GFP at MOI=10 and mock transduction (FreeStyle™ 293 Expression Medium) were used as controls (**Figure 1A**). Cell pellets and culture supernatants were collected 48 and 72 hours post transduction for further analysis.

Cytotoxicity was assessed using the LDH-Glo™ assay (Promega, J2380/J2381) per the manufacturer’s instructions (12). An aliquot of culture media from mock transduction control received 40 µL of 10% Triton X-100 and was incubated at room temperature for a minimum of 15 minutes. Cellular supernatants for the assessment of lactate dehydrogenase (LDH) were diluted 1:20 in LDH storage buffer [200 mM Tris-HCl (pH = 7.3); glycerol 10%; bovine serum albumin 1%]. Supernatant from Triton-X-100-treated cells was serially diluted to within the linear range of the assay. Diluted samples were combined 1:1 with detection reagent (LDH Detection Enzyme Mix with Reductase Substrate) and added to a 384-well plate in duplicate. A standard curve was prepared from the positive control and added to the plate in triplicate. Samples were analyzed using an EnVision^®^ plate reader (PerkinElmer) following a 50-minute incubation at room temperature.

#### SARS-CoV-2 S1 and S2 Subunit Western Blot and ELISA

Cell pellets were examined for expression of spike protein by Western blot. Cell pellet (10 μg protein per lane) samples were run on sodium dodecyl sulphate–polyacrylamide gel electrophoresis (SDS-PAGE) gels and then transferred onto polyvinylidene fluoride (PVDF) membranes using the iBlot^®^ 2 dry blotting system (Thermo Fisher Scientific). PVDF membranes were blocked, washed and incubated with primary and secondary antibodies using an iBind™ (Thermo Fisher Scientific) system. Anti-SARS-CoV-2/2019-n-CoV Spike receptor binding domain (RBD) and Spike S2 (Sino Biological, 40592-T62 and 40590-T62) were used as primary antibodies. Anti-β-Actin (Sigma, A1978) was used as a loading control. IRDye 680CW (Licor, 926-68073) and IRDye 800CW (Licor, 926-32212) were used as secondary antibodies. Fluorescence was visualized using an Odyssey CLx imager (Li-Cor Biosciences).

Spike protein S1 and S2 subunit expression levels in culture supernatant were measured by ELISA using 2130-wt or 2196-wt, two monoclonal SARS-CoV-2 spike protein RBD neutralizing antibodies (13), at 100 μg/mL as capture antibodies. 96-well high-binding plates were coated with 100 μg/mL 2130-wt or 2196-wt in 1X PBS at 100 μL per well and incubated at 40°C overnight. Wells were washed 4 times with Blocker™ Casein phosphate buffered saline (PBS) buffer (Thermo 37528) and blocked with 200 μL per well of casein for 1 hour at room temperature. For spike protein standard curve, purified SARS-CoV-2 S trimer was serially diluted 1:3 beginning with high concentration of 3 μg/mL down to 0.001 ng/mL in casein. Samples were tested undiluted and at 1:5 dilutions in casein. Standards and samples were added to wells at 100 μL per well and incubated for 1 hour at room temperature. Wells were washed 4 times with 300 μL per well of casein. Next, secondary antibodies (anti-mouse HRP [Dako P0447] or anti-rabbit HRP (Cell Signaling, 7074S) were diluted 1:10,000 in casein and added to wells at 100 μL per well and incubated for 1 hour at room temperature. Wells were again washed four times with 300 μL per well of casein. 3,3,5,5-Tetramethylbenzidine (TMB) KPL SureBlue (SeraCare, 5120-0074) (equilibrated to room temperature) was added to wells at 100 μL per well and incubated in the dark at room temperature for 5–10 minutes. Reactions were stopped by adding 2N H_2_SO_4_ at 100 μL per well. Plates were analyzed with an EnVision^®^ plate reader (PerkinElmer) to read absorbance at 450 nm.

### *In Vivo* Animal Procedures and Study Design

#### Animals

All *in vivo* experimental procedures were approved by the Home Office, United Kingdom, with adherence to the Animals (Scientific Procedures) Act 1986. The regulations conform to EU Directive 2010/63/EU and achieve the standard of care required by the US Department of Health and Human Services’ Guide for the Care and Use of Laboratory Animals. Animal studies were conducted according to Good Laboratory Practice regulations for nonclinical laboratory studies and complied with ARRIVE guidelines.

Animal procedures used equal numbers of male and female CD-1 mice aged 8–12 weeks and weighing 20–50 g at the time of dosing. Animals were obtained from Charles River Laboratories (Charles River UK Limited). Mice were examined prior to allocation to study stock. Mice were excluded if they presented with lesions, masses, and/or swellings upon initial examination. Males and females were randomized separately.

Males were housed individually, while females were housed at 2–3 mice per cage. The targeted conditions for animal room environment were 19–23°C, 40–70% humidity, ventilated with >10 air changes per hour, and with a 12-hour light/dark cycle unless interrupted by study procedures/activities. SDS Rat and Mouse No. 1 Diet SQC Expanded, and water, were provided *ad libitum* throughout the study, except during designated procedures.

#### Test Agent

AZD1222 (MS00684-92) with a virus particle concentration of 2.13 x10^12^/mL was used as the test agent. For control experiments, a buffer (vehicle) of 10 mM histidine, 7.5% (v/w) sucrose, 35 mM sodium chloride, 1 mM magnesium chloride, 0.1% (v/w) Polysorbate-80, 0.1 mM ethylenediaminetetraacetic acid (EDTA), and 0.5% (v/w) ethanol, pH 6.6, was used.

#### Study Design

This study was performed unblinded. Mice were randomly assigned to receive control (n = 12) or AZD1222 *via* intravenous (IV) (n=48) or intramuscular (IM) (n = 48) injection. Mice assigned to the IV route-of-administration group received AZD1222 at a concentration of 1 x10^9^ viral particles (VP) by single injection at a fixed volume of 30 µL on Day 1. As this was the first time AZD1222 was administrated by IV dosing, mice were split into three batches of increasing size, and 1-day pauses were added between dosing batches 1, 2, and 3 to ensure the tolerability of test agent before dosing larger cohorts of animals. Mice assigned to the IM route-of-administration group received AZD1222 at a concentration of 1x10^10^ VP by single injection of a fixed volume of 30 µL to the right hind limb (thigh) on Day 1.

#### In Life Procedures and Assessments

Mortality/moribundity were checked throughout the study at the beginning and end of the working day. All mice received at least one physical examination during the pre-treatment portion of the study. Mice were examined regularly throughout the day post dosing for potential reactions to AZD1222 or control, with particular attention paid to the mice during and for the first hour after dosing.

Body weights were collected as deemed necessary by the technical staff for welfare purposes only. Therefore, due to the lack of concurrent data, body weights were compared to pretreatment values and no conclusions are drawn from this dataset. In animals dosed with AZD1222 IV at 1x10^9^ VP a decrease in body weight was observed at various timepoints throughout the first week of this study. There were no changes in body weight in animals dosed with AZD1222 IM at 1x10^10^ VP.

#### Blood Sample Collection and Storage

Blood samples were collected from the orbital sinus following non-recoverable isoflurane anesthesia for serum biomarker bioanalysis and assessment of hematology parameters (**Supplemental Table 1**). Blood samples for hematology assessments were combined with K_2_EDTA anticoagulant. Serum samples for S1 sequential sandwich electrochemiluminescence immunoassay were allowed to clot at ambient temperature for ≥60 mins before centrifugation at 1500 x g for 10 mins at 4°C. Resultant serum was separated and stored at –80°C prior to analysis.

#### Serum S1 Sequential Sandwich Electrochemiluminescence (ECL) Immunoassay

S1 subunit levels in serum were assessed using a validated immunoassay. MSD 96-well small spot streptavidin plates were coated with biotinylated SARS-CoV-2 spike capture antibody (MSD, C20ADB-3) and incubated overnight at 2–8°C. Wells were washed three times with 1x Tris buffer. MSD diluent 11 was added to the wells and allowed to incubate for 30 mins at 25°C. Standard curve and samples were added to wells and allowed to incubate for 120 mins at 25°C. Wells were washed three times with 1x Tris buffer. Detection reagent was added to the wells and allowed to incubate for 60 mins at 25°C. Wells were washed for a final three times with 1x Tris buffer prior to the addition of MSD Gold Read buffer. Spike protein was detected using SULFO-TAG SARS-CoV-2 Spike detection antibody (MSD, D20ADB-3). Plates were analyzed using a MSD S600 Meso Sector Imager Microplate Reader within 10 mins of the addition of read buffer. The lower limit of quantification for the assay was 6.30 pg/mL relative to the SARS-CoV-2 calibrator (MSD, C00ADB-2).

#### Assessment of Effects on Hematology Parameters

Changes in hematology parameters were assessed based on reference ranges observed in mice under similar study conditions at concurrent and non-concurrent timepoints from historical control data for the testing facility. Group mean values were determined for each timepoint post-vaccination and compared to the reference range to assess for any potential AZD1222-related changes.

#### Histology, Histopathology, and Immunohistochemistry

Samples of injection site, spleen and bone marrow (sternum and femora-tibial joint) from animals sacrificed at Day 14 post IM injection were fixed in 10% neutral-buffered formalin and processed to paraffin blocks using routine methods. Tissues were sectioned at 4 µm thickness and stained with an immunohistochemical method using a rabbit monoclonal antibody specific to the SARS-Cov2 spike protein (E5S3V, Cell Signaling Technology) at 0.1 µg/ml dilution, on an automated Bond-RX immunostainer (Leica Biosystems), using DAB as a chromogen. Whole slide images were obtained using an Aperio scanner (Leica Biosystems), and were examined by a board-certified veterinary pathologist.

#### Statistical Analyses

A formal power analysis was considered inappropriate due to the exploratory nature of this study. Three male and three female mice were used per timepoint per vaccination group to ensure reliability of the toxicokinetic and tolerability estimates. Means and standard deviations were calculated where appropriate.

## Results

### S1 Subunit Is Cleaved *In Vitro* Following AZD1222 Transduction of HEK293x Cells

We assessed the impact of SARS-CoV-2 transgene expression on HEK293x cytotoxicity. Minor levels of cytotoxicity were expected as HEK293x cells are permissive to adenovector propagation by virtue of expressing the adenovirus E1A and E1B genes in *trans* (14), thus incurring the lytic portion of the late-stage adenovirus replication cycle (15). AZD1222-induced cytotoxicity was greatest with MOI=1 and MOI=3 (both 5.5%) at 48 hours post-transduction and with MOI=1 (23.6%) at 72 hours post-transduction (**Figure 1A**, **Supplemental Table 2**). Cell death was not a result of the spike transgene expression as cytotoxicity was also observed 48 hours (12.6%) and 72 hours (19.5%) post transduction with ChAdOx-1-GFP at MOI=10.

Presence of full-length spike protein in cell pellets was confirmed by detection of S1 and S2 subunits by Western blot (**Figure 1B**). S1 and S2 subunit expression was absent in cells transduced with ChAdOx1-GFP or non-transduced controls (**Figures 1B, C**). We observed the presence of cleaved S1 subunit in culture supernatant at 48 and 72 hours following AZD1222 transduction (**Figure 1D**). Higher S1 subunit levels were observed in cells transduced with lower MOIs at 72 hours, perhaps due to more efficient production of spike protein, or due to lesser cytotoxicity, with lower virus-to-cell ratios. Full-length spike protein was not observed in the supernatant at either 48- or 72-hours post-transduction (**Figure 1E**). Similar results were observed with Western blots of culture supernatants (data not shown).

### S1 Subunit Is Cleaved and Rapidly Cleared *In Vivo* Following IM or IV AZD1222 Immunization With Similar Kinetics

**Figure 2:**
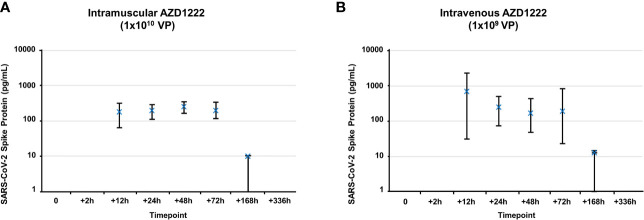
Kinetics of S1 subunit detection following AZD1222 immunization. Levels of SARS-CoV-2 spike protein S1 subunit detected in serum following IM **(A)** or IV **(B)** AZD1222 immunization. Data points represent mean SARS-CoV-2 Spike protein concentrations observed in male and female mice. Error bars indicate minimum and maximum concentrations per timepoint.

We next assessed whether S1 subunit cleavage occurs *in vivo* using CD-1 mice. Mice received higher doses of AZD1222 (per dose/weight ratio) than those in clinical use (7) to maximize the potential for detecting cleaved S1 subunit and to evaluate the effects of exaggerated AZD1222 pharmacology. S1 subunit was detectable in murine sera 12 hours post IM immunization (**Figure 2A**). Mean soluble S1 subunit protein levels decreased within 3 days (**Table 1**) and were below the limit of quantification in 4/6 samples at 7 days and in 6/6 samples at 14 days post-IM immunization (**Supplemental Table 3**). IV immunization produced higher mean levels of soluble S1 subunit protein with similar expression kinetics to IM immunization (**Figure 2B**; **Table 1**; **Supplemental Table 3**).

**Table 1:** Mean SARS-CoV-2 soluble S1 subunit levels post-AZD1222 immunization (pg/mL).

	Males		Females	
Total Viral Particle (VP)/Dose	1x10^9^	1x10^10^	1x10^9^	1x10^10^
Hours post-AZD1222 immunization	IV	IM	IV	IM
0	BLQ	BLQ	BLQ	BLQ
2 hours	BLQ	BLQ	BLQ	BLQ
12 hours	356.0	233.0	1043.0	126.1
24 hours	244.0	206.3	249.0	187.0
48 hours	165.6	276.3	208.7	233.0
72 hours	325.3	166.7	55.2	227.3
168 hours	13.0	10.2	BLQ	10.6
336 hours	BLQ	BLQ	BLQ	BLQ

BLQ, below the limit of quantification (6.3 pg/mL); IM, intramuscular; IV, intravenous; VP, viral particles.

It is possible that absence of S1 subunit detection after 14 days is due to formation of host anti-S1-subunit antibodies, which would inhibit detection by serum immunoassay. However, immunohistochemistry analyses on IM injection sites revealed no significant expression of spike protein 14 days post AZD1222 immunization (**Supplemental Figure 1**). Bone marrow samples from all animals contained abundant amounts of hematopoietic cells of various lineages, including megakaryocytes. Spleen samples contained variable numbers of hematopoietic cells, as is common in mice. Although another study observed persistence of AZD1222 at IM injection sites by quantitative polymerase chain reaction 29 days post-immunization (16), none of the tissue samples showed evidence of spike protein. Sections of blood vessels did not show evidence of intravascular positive staining in circulating cells including platelets.

### AZD1222 Induced Modest/Transient Changes to Host Hematology Parameters Immediately Following Immunization

The effects of AZD1222 vaccination on host hematology parameters and initial immune responses were assessed through evaluating platelet and total white blood cell (consisting of lower lymphocytes, monocytes, and/or eosinophils) counts immediately following immunization (**Table 2**). IM administration of AZD1222 resulted in transiently lower platelets (~73% of vehicle mean; 12 hours), and lower total white blood cells (12–24 hours) counts post immunization. IV administration resulted in lower platelets (69–84% of vehicle mean; 24–72 hours) and lower white blood cells (12–24 hours) post immunization. Neutrophils were transiently higher 2 hours post IV immunization. Hematology parameters at other timepoints were considered unrelated to AZD1222 and were attributed to biological variation, as similar variations were seen in vehicle control and/or were of a magnitude of change commonly observed in mice under similar study conditions at concurrent and non-concurrent timepoints, or within historical control data for the testing facility.

**Table 2:** Effects on host immune response and hematology parameters immediately following AZD1222 immunization.

	Platelets (10^9^/L)			Total White Blood Cells (10^9^/L)			Neutrophils (10^9^/L)		
Hours post-AZD1222 immunization	Vehicle	IV 1x10^9^ VP	IM 1x10^10^ VP	Vehicle	IV 1x10^9^ VP	IM 1x10^10^ VP	Vehicle	IV 1x10^9^ VP	IM 1x10^10^ VP
0	1033.0	–	–	6.933	–	–	0.840	–	–
2	NA	–	–	NA	–	–	NA	2.940↑	–
12	NA	–	753.5**↓**	NA	1.593↓	1.040**↓**	NA	–	–
24	NA	868.0**↓**	–	NA	1.913**↓**	3.267**↓**	NA	–	–
48	NA	721.3**↓**	–	NA	–	–	NA	–	–
72	NA	815.3**↓**	–	NA	–	–	NA	–	–
166	NA	–	–	NA	–	–	NA	–	–
336	1295.0	–	–	6.977	–	–	0.890	–	–

Values presented are group mean absolute values that were outside of the reference ranges observed in mice under similar study conditions at concurrent and non-concurrent timepoints from historical control data for the testing facility.

IM, intramuscular; IV, intravenous; NA, Not applicable; VP, viral particles; – = Group mean value was within the reference range, indicating no AZD1222-related change; **↓ = **decreased versus reference range; ↑ = increased versus reference range.

## Discussion

The COVID-19 pandemic continues to cause substantial global morbidity and mortality, with an estimated 28 million life-years lost in 2020 (17). Gene-based vaccines that elicit production of the spike protein in vaccinees have substantially reduced the risk of severe disease and death from COVID-19 (7–10), and are an invaluable tool for mitigating future SARS-CoV-2 outbreaks (18, 19). *In vitro* studies of SARS-CoV-2 have suggested that the spike protein is directly responsible for mediating the thromboembolic complications observed during severe COVID-19 (2, 20, 21). Therefore, it was important to evaluate the effects of COVID-19 vaccine-induced spike protein on host immune and hematologic parameters immediately following immunization.

Within this manuscript we demonstrate that AZD1222-induced S1 subunit is cleaved *in vitro* and *in vivo*. S1 subunit cleavage is likely the result of host proteolytic cleavage [e.g., *via* transmembrane serine protease 2, cathepsin or furin (22, 23)] rather than due to adenovirus-induced cell death (15), as suggested by the low cytotoxicity and lack of S2 subunit detection following AZD1222 transduction. We also demonstrated that, following cleavage, the S1 subunit is rapidly cleared and is no longer detectable from 7–14 days following either IM or IV immunization. Similar quantities of S1 subunit have also been observed in individuals with severe COVID-19 (4). We also observe similar expression kinetics to those observed following IM immunization with mRNA-1273 COVID-19 vaccine (24). Cleavage of vaccine-induced spike protein from host cells may complement other modes of host cell secretion (e.g., endosomal secretion) and facilitate subsequent processing by antigen-presenting cells and the initiation of adaptive immune responses (25).

We observed that IM vaccination induced modest/transient changes to host platelet counts 12 hours following immunization, with similar scale decreases observed 24–72 hours following IV immunization. Murine models of thrombocytopenia (26), with demonstrated physiological relevance to human platelet count/function, suggest that the transient platelet reductions observed following AZD1222 vaccination should not affect host thrombosis or hemostasis. Total white blood cell counts were decreased within the reference range for leukopenia (i.e., <2.0x10^9^ total white blood cells per liter) (27) 12 hours following IM and IV immunization but increased to within normal reference ranges (i.e., 2.0–10.0x10^9^ total white blood cells per liter) by 24 hours following IM immunization. Transient leukopenia is a characteristic sign of early responses to viral infections and is routinely documented following vaccination (28–33). Following immunization, AZD1222 enters host cells *via* the widely expressed coxsackie and adenovirus receptor (34), wherein detection of AZD1222-derived viral nucleic acids by host cell pathogen recognition receptors initiates production of pro-inflammatory cytokines, chemokines, and type I interferons (35). Neutrophil cell counts were increased 2 hours following IV vaccination, corresponding to expected initial cytokine and chemokine responses to adenoviral infection (36). The initial innate immune response also attracts antigen-presenting cells to the site of immunization, facilitating an induction of S glycoprotein-specific CD8+ and CD4+ T helper 1 T cells, and antibodies that have been observed from 14 days post-immunization in other murine studies of AZD1222 (37–41). Platelet, total white blood cell, and neutrophil counts were unchanged at subsequent timepoints, suggesting that the changes elicited by AZD1222 vaccination are transient and quickly resolved following immunization.

It is important to note that we observed S1 subunit cleavage *in vivo* using AZD1222 concentrations ~30 (IV dose; 1x10^9^ VP) to ~300 (IM dose; 1x10^10^ VP) times greater than in current clinical use (based on dose/weight ratio (7)) and therefore are assessing the effects of exaggerated AZD1222 pharmacology within these experiments. Additionally, a limitation of our study is the distinct differences in COVID-19 pathophysiology between humans and murine models, which may limit the wider interpretation of our findings (42). Although, the SARS-CoV-2 virus can be adapted to improve spike protein binding to the murine ACE2 receptor by serial passage or by using reverse genetics to improve RBD-murine ACE2 receptor binding (43, 44), these adapted viruses still only confer mild forms of COVID-19 disease. Thus, it would be interesting to explore the kinetics and implications of S1 cleavage, and potential effects of AZD1222 and other COVID-19 vaccines on host hematology parameters immediately following vaccination in other animal models (e.g., non-human primates, Syrian hamsters) that may better reflect COVID-19 disease. K18-hACE2 is a transgenic mouse strain that expresses a human ACE2 receptor driven by the cytokeratin-18 (K18) gene promotor, and that has been observed to succumb to SARS-CoV-2 infection due to lung and brain pathology from severe lethal cytokine storm 4–6 days post-SARS-CoV-2 challenge (45, 46). Recombinant SARS-CoV-2 spike protein has been observed to directly bind K18-hACE2 platelets and potentiate thrombosis formation in wild-type mice following K18-hACE2 platelets transfusion (2), albeit using concentrations of spike protein that greatly exceed the concentration of S1 subunit observed in sera of individuals with COVID-19 (4) or following mRNA-1273 vaccination (24). It not yet known whether this finding can be replicated with ‘live’ SARS-CoV-2 virus or whether spike protein can be induced in sufficient quantities by COVID-19 vaccines to produce a similar result. However, insights from this model may prove invaluable for exploring the etiology of the rare hematologic and vascular complications following COVID-19 vaccination (3).

In conclusion, our results provide further insight to the host response to AZD1222 vaccination. We demonstrate the cleavage of vaccine-induced SARS-CoV-2 spike protein S1 subunit *in vitro* and *in vivo* following IM and IV immunization using concentrations several magnitudes higher than currently used in humans, without deleterious effects on the host. It is unlikely that any potential adverse effects following AZD1222 vaccination can be attributed to persistent S1 subunit expression as this protein is no longer detectable in host sera by 14 days post-vaccination. We also describe transient and quickly resolved effects on host blood parameters immediately following AZD1222 immunization. Collectively these findings, alongside data from pivotal Phase 3 studies (7, 47) and ongoing pharmacosurveillance, support the continued use of AZD1222 to mitigate the COVID-19 pandemic.

## Data Availability Statement

Data associated with this study are available in the main text or the **Supplementary Materials**.

## Ethics Statement

The animal study was reviewed and approved by the Home Office, United Kingdom, with adherence to the Animals (Scientific Procedures) Act 1986. The regulations conform to EU Directive 2010/63/EU and achieve the standard of care required by the US Department of Health and Human Services’ Guide for the Care and Use of Laboratory Animals. Animal studies were conducted according to Good Laboratory Practice regulations for nonclinical laboratory studies and complied with ARRIVE guidelines.

## Author Contributions

Study design: RS and TC. HEK 293x transduction: TB. LDH assay: TB. Western blots: VT. ELISA: VT. *In vivo* study monitor: GA and SM. Assessment of hematology parameters: PC. Serum biomarker analysis: JG, CJ, CMT, and VS. Immunohistochemistry experiments and analysis: AD and JML. Critical revision of the manuscript and approval of the final draft: COH under direction of all authors. Confirmation of data accuracy and approval of the final draft: All authors.

## Funding

This research was funded by AstraZeneca.

## Conflict of Interest

AD is an employee of LabCorp Early Development Laboratories Limited, a contract research organization that supported AstraZeneca with immunohistochemistry analyses during this investigation. All other authors report a relationship with AstraZeneca that includes employment and stock options.

This study received funding from AstraZeneca. The funder had the following involvement with the study: study design; collection, analysis and interpretation of data; the writing of this article and the decision to submit it for publication.

## Publisher’s Note

All claims expressed in this article are solely those of the authors and do not necessarily represent those of their affiliated organizations, or those of the publisher, the editors and the reviewers. Any product that may be evaluated in this article, or claim that may be made by its manufacturer, is not guaranteed or endorsed by the publisher.
