# Supplementary material for: SARS-CoV-2 Spike Protein Expression In Vitro and Hematologic Effects in Mice Vaccinated With AZD1222 (ChAdOx1 nCoV-19)
Source: Front Immunol. 2022 Apr 12;13:836492. doi: 10.3389/fimmu.2022.836492 (PMC9039667; doi:10.3389/fimmu.2022.836492)
Supplement: Supplementary file 1 [file Image_1.pdf]

**Supplemental Figures:**

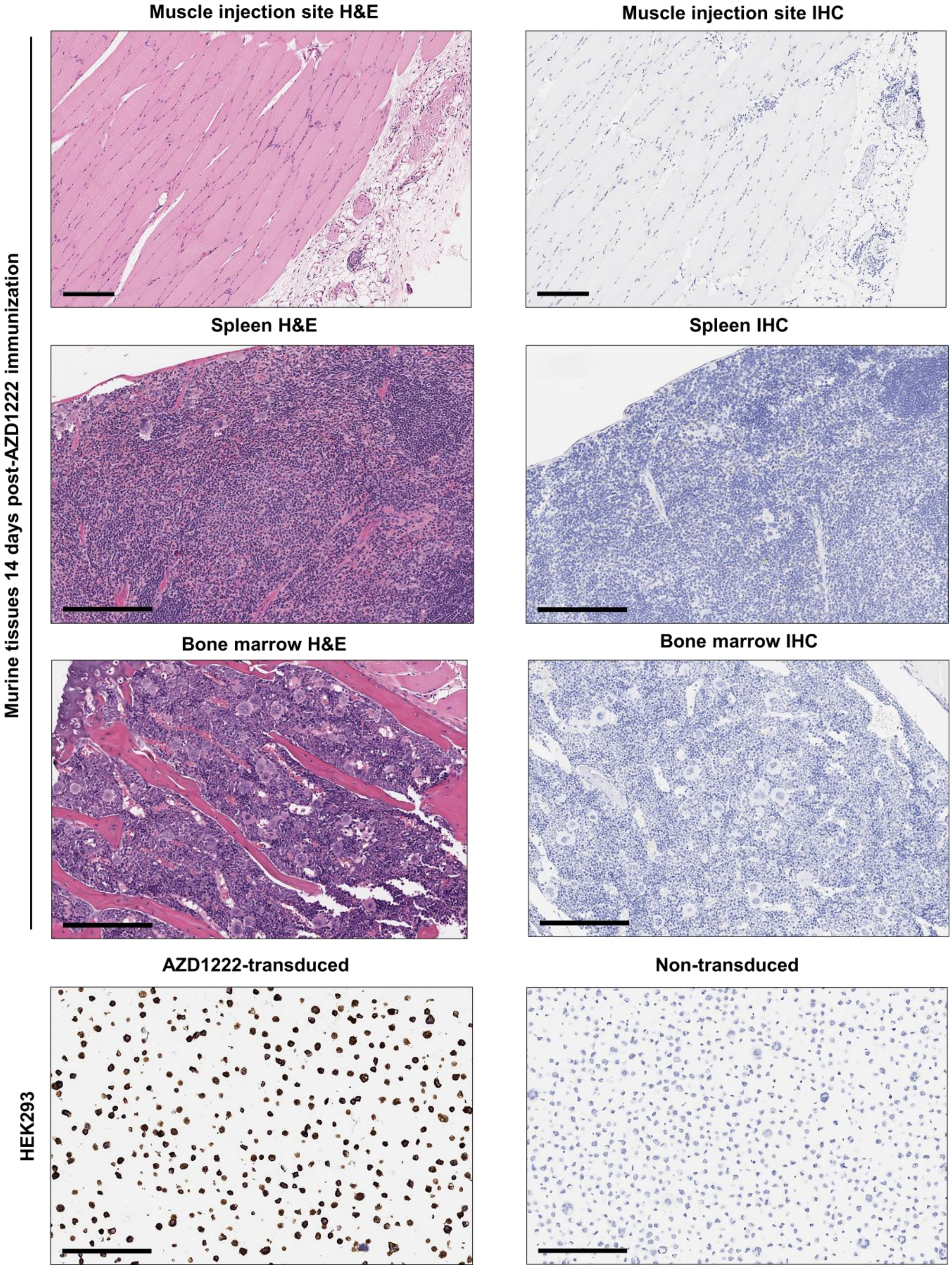




This is a provisional file, not the final typeset article

**Supplemental Figure 1. SARS-CoV-2 spike protein is no longer detectable via immunohistochemistry 14 days post-AZD1222 immunization**

Muscle injection sites, spleen and bone marrow were evaluated 14 days post AZD1222 immunization by hematoxylin and eosin and immunohistochemistry. HEK293x cells transduced with AZD1222 were used as positive controls for bone marrow and splenic analyses due to negative injection site staining. Scale bar represents 200 µm.

H&E, Hematoxylin and eosin; IHC, immunohistochemistry.
