# Supplementary material for: SARS-CoV-2 Spike Protein Expression In Vitro and Hematologic Effects in Mice Vaccinated With AZD1222 (ChAdOx1 nCoV-19)
Source: Front Immunol. 2022 Apr 12;13:836492. doi: 10.3389/fimmu.2022.836492 (PMC9039667; doi:10.3389/fimmu.2022.836492)
Supplement: Supplementary file 2 [file Table_1.docx]

**Supplemental Tables**

| Red blood cell count  Hemoglobin  Hematocrit  Mean corpuscular volume  Mean corpuscular hemoglobin  Mean corpuscular hemoglobin concentration  Red blood cell distribution width  Platelet count  Mean platelet volume  Mean platelet dry mass  Reticulocyte count (absolute)  Reticulocytes (%)  Mean platelet component  Plateletcrit  Platelet distribution width (PDW)  Platelet component distribution width  Platelet mass distribution width | White blood cell count  Neutrophil count (absolute)  Lymphocyte count (absolute)  Monocyte count (absolute)  Eosinophil count (absolute)  Basophil count (absolute)  Large unstained cells (absolute)  Blood smear |
| --- | --- |

**Supplemental Table 1. Hematology parameters assessed per protocol**
