# Supplementary material for: SARS-CoV-2 Spike Protein Expression In Vitro and Hematologic Effects in Mice Vaccinated With AZD1222 (ChAdOx1 nCoV-19)
Source: Front Immunol. 2022 Apr 12;13:836492. doi: 10.3389/fimmu.2022.836492 (PMC9039667; doi:10.3389/fimmu.2022.836492)
Supplement: Supplementary file 3 [file Table_2.docx]

|  |  | | | |
| --- | --- | --- | --- | --- |
| Hours Post-Transduction | Sample | Concentration ± SD (mU/mL) | %CV | % Cytotoxicity ± SD |
| 48 hours | Mock | 591.5 ± 96.2 | 16.3 | 0.0^a^ |
|  | AZD1222 MOI=0.1 | 390.4 ± 111.9 | 28.7 | -1.0 ± 1.1 |
|  | AZD1222 MOI=0.3 | 981.7 ± 191.0 | 19.5 | 1.9 ± 0.6 |
|  | AZD1222 MOI=1 | 1743.9 ± 404.3 | 23.2 | 5.5 ± 1.8 |
|  | AZD1222 MOI=3 | 1749.4 ± 671.7 | 38.4 | 5.5 ± 3.1 |
|  | AZD1222 MOI=10 | 1648.2 ± 251.3 | 15.2 | 5.0 ± 1.0 |
|  | ChAdOx1-GFP MOI=10 | 3246.8 ± 605.8 | 18.7 | 12.6 ± 3.2 |
|  | Mock Triton X-100 | 21603.8 ± 1179.6 | 5.5 | 100.0^a^ |
| 72 hours | Mock | 1155.9 ± 38.8 | 3.4 | 0.0^a^ |
|  | AZD1222 MOI=0.1 | 2488.7 ± 452.0 | 18.2 | 4.8 ± 2.1 |
|  | AZD1222 MOI=0.3 | 3858.3 ± 403.7 | 10.5 | 9.7 ± 0.7 |
|  | AZD1222 MOI=1 | 7707.2 ± 506.3 | 6.6 | 23.6 ± 3.5 |
|  | AZD1222 MOI=3 | 5885.5 ± 922.7 | 15.7 | 17.0 ± 2.1 |
|  | AZD1222 MOI=10 | 3859.3 ± 58.4 | 1.5 | 9.7 ± 1.0 |
|  | ChAdOx1-GFP MOI=10 | 6583.9 ± 881.8 | 13.4 | 19.5 ± 1.8 |
|  | Mock Triton X-100 | 28964.6 ± 1823.8 | 6.3 | 100.0^a^ |

**Supplemental Table 2. LDH concentration and % cytotoxicity by sample**

^a^Mock and triton-treated samples were the reference values for determining % cytotoxicity; all mock replicates had calculated % cytotoxicity of 0 and all mock triton-treated replicates had calculated % cytotoxicity of 100. CV (%), coefficient of variation (precision); MOI, multiplicity of infection; mU/mL, milliunit per milliliter; SD, standard deviation.
