# Supplementary material for: SARS-CoV-2 Spike Protein Expression In Vitro and Hematologic Effects in Mice Vaccinated With AZD1222 (ChAdOx1 nCoV-19)
Source: Front Immunol. 2022 Apr 12;13:836492. doi: 10.3389/fimmu.2022.836492 (PMC9039667; doi:10.3389/fimmu.2022.836492)
Supplement: Supplementary file 4 [file Table_3.docx]

| **Test agent** | **Timepoint (Hours  Post-AZD122 Immunization)** | **Replicate 1** | **Replicate 2** | **Mean S1 subunit (pg/mL)** | **% CV** |
| --- | --- | --- | --- | --- | --- |
| Vehicle | 0 | BLQ | BLQ | BLQ | NA |
|  |  | BLQ | BLQ | BLQ | NA |
|  |  | BLQ | BLQ | BLQ | NA |
|  |  | BLQ | BLQ | BLQ | NA |
|  |  | BLQ | BLQ | BLQ | NA |
|  |  | BLQ | BLQ | BLQ | NA |
|  | 336 | BLQ | BLQ | BLQ | NA |
|  |  | BLQ | BLQ | BLQ | NA |
|  |  | BLQ | BLQ | BLQ | NA |
|  |  | BLQ | BLQ | BLQ | NA |
|  |  | BLQ | BLQ | BLQ | NA |
|  |  | BLQ | BLQ | BLQ | NA |
| AZD1222  1x10^9^  (IV) | 0 | BLQ | BLQ | BLQ | NA |
|  |  | BLQ | BLQ | BLQ | NA |
|  |  | BLQ | BLQ | BLQ | NA |
|  |  | BLQ | BLQ | BLQ | NA |
|  |  | BLQ | BLQ | BLQ | NA |
|  |  | BLQ | BLQ | BLQ | NA |
|  | 2 | BLQ | BLQ | BLQ | NA |
|  |  | BLQ | BLQ | BLQ | NA |
|  |  | BLQ | BLQ | BLQ | NA |
|  |  | BLQ | BLQ | BLQ | NA |
|  |  | BLQ | BLQ | BLQ | NA |
|  |  | BLQ | BLQ | BLQ | NA |
|  | 12 | 742 | 711 | 727 | 3.0 |
|  |  | 31.8 | 32.4 | 32.1 | 1.2 |
|  |  | 315 | 302 | 309 | 3.1 |
|  |  | 602 | 652 | 627 | 5.6 |
|  |  | 2342 | 2254 | 2298 | 2.7 |
|  |  | 205 | 203 | 204 | 0.5 |
|  | 24 | 203 | 196 | 200 | 2.6 |
|  |  | 340 | 343 | 341 | 0.6 |
|  |  | 180 | 203 | 191 | 8.4 |
|  |  | 178 | 197 | 187 | 6.8 |
|  |  | 73.2 | 72.6 | 72.9 | 0.6 |
|  |  | 500 | 475 | 487 | 3.6 |
|  | 48 | 77.6 | 72.4 | 75.0 | 4.9 |
|  |  | 289 | 278 | 283 | 2.9 |
|  |  | 48.9 | 47.5 | 48.2 | 2.0 |
|  |  | 86.8 | 85.3 | 86.1 | 1.3 |
|  |  | 431 | 438 | 435 | 1.2 |
|  |  | 102 | 108 | 105 | 3.8 |
|  | 72 | 119 | 121 | 120 | 1.0 |
|  |  | 832 | 830 | 831 | 0.2 |
|  |  | 24.7 | 24.8 | 24.8 | 0.2 |
|  |  | 23.0 | 23.3 | 23.2 | 1.1 |
|  |  | 104 | 96.3 | 100 | 5.2 |
|  |  | 40.9 | 43.8 | 42.4 | 4.9 |
|  | 168 | 11.7 | 11.6 | 11.6 | 0.7 |
|  |  | 13.8 | 14.7 | 14.3 | 4.6 |
|  |  | BLQ | BLQ | BLQ | NA |
|  |  | BLQ | BLQ | BLQ | NA |
|  |  | BLQ | BLQ | BLQ | NA |
|  |  | BLQ | BLQ | BLQ | NA |
|  | 336 | BLQ | BLQ | BLQ | NA |
|  |  | BLQ | BLQ | BLQ | NA |
|  |  | BLQ | BLQ | BLQ | NA |
|  |  | BLQ | BLQ | BLQ | NA |
|  |  | BLQ | BLQ | BLQ | NA |
|  |  | BLQ | BLQ | BLQ | NA |
| AZD1222  1x10^10^  (IM) | 0 | BLQ | BLQ | BLQ | NA |
|  |  | BLQ | BLQ | BLQ | NA |
|  |  | BLQ | BLQ | BLQ | NA |
|  |  | BLQ | BLQ | BLQ | NA |
|  |  | BLQ | BLQ | BLQ | NA |
|  |  | BLQ | BLQ | BLQ | NA |
|  | 2 | BLQ | BLQ | BLQ | NA |
|  |  | BLQ | BLQ | BLQ | NA |
|  |  | BLQ | BLQ | BLQ | NA |
|  |  | BLQ | BLQ | BLQ | NA |
|  |  | BLQ | BLQ | BLQ | NA |
|  |  | BLQ | BLQ | BLQ | NA |
|  | 12 | 322 | 302 | 312 | 4.6 |
|  |  | 142 | 128 | 135 | 7.6 |
|  |  | 265 | 240 | 252 | 7.1 |
|  |  | 210 | 197 | 203 | 4.5 |
|  |  | 68.7 | 62.1 | 65.4 | 7.1 |
|  |  | 116 | 103 | 110 | 8.2 |
|  | 24 | 258 | 239 | 248 | 5.5 |
|  |  | 257 | 227 | 242 | 8.6 |
|  |  | 134 | 124 | 129 | 5.2 |
|  |  | 313 | 277 | 295 | 8.6 |
|  |  | 115 | 106 | 111 | 5.9 |
|  |  | 149 | 161 | 115 | 5.5 |
|  | 48 | 321 | 297 | 309 | 5.4 |
|  |  | 268 | 251 | 259 | 4.6 |
|  |  | 269 | 253 | 261 | 4.2 |
|  |  | 177 | 180 | 179 | 1.0 |
|  |  | 369 | 333 | 351 | 7.2 |
|  |  | 187 | 153 | 170 | 14.2 |
|  | 72 | 376 | 328 | 352 | 9.8 |
|  |  | 227 | 194 | 211 | 11.1 |
|  |  | 187 | 180 | 184 | 2.9 |
|  |  | 155 | 161 | 158 | 2.9 |
|  |  | 159 | 156 | 158 | 1.6 |
|  |  | 120 | 118 | 119 | 1.3 |
|  | 168 | 10.5 | 9.81 | 10.2 | 4.9 |
|  |  | BLQ | BLQ | BLQ | NA |
|  |  | BLQ | BLQ | BLQ | NA |
|  |  | BLQ | BLQ | BLQ | NA |
|  |  | 10.8 | 10.4 | 10.6 | 2.6 |
|  |  | BLQ | BLQ | BLQ | NA |
|  | 336 | BLQ | BLQ | BLQ | NA |
|  |  | BLQ | BLQ | BLQ | NA |
|  |  | BLQ | BLQ | BLQ | NA |
|  |  | BLQ | BLQ | BLQ | NA |
|  |  | BLQ | BLQ | BLQ | NA |
|  |  | BLQ | BLQ | BLQ | NA |

**Supplemental Table 3. SARS-CoV-2 S1 subunit concentrations in serum, by individual animal**

BLQ indicates values below lower limit of quantification (6.30 pg/mL). BLQ, below lower limit of quantification; CV (%) coefficient of variation (precision);
NA, not applicable.
